# Gender Confirmation Surgery: An Update for the Primary Care Provider

**DOI:** 10.1089/trgh.2015.0006

**Published:** 2016-01-01

**Authors:** Loren S. Schechter

**Affiliations:** University Plastic Surgery, Morton Grove, Illinois.

**Keywords:** gender confirmation surgery, gender nonconforming, metoidioplasty, phalloplasty, transgender, vaginoplasty

## Abstract

Increased advocacy efforts and expanded third-party insurance coverage have improved access to healthcare for transgender individuals. In conjunction with mental health and medical professionals, gender surgeries offer an important step in allowing individuals to realize their true selves. To provide quality multidisciplinary care, primary care doctors need to understand challenges facing transgender individuals and treatment options available to them. In this article, we will review the role of the surgeon and the goals of various gender confirming surgeries. In addition, we will provide an overview of the available surgical options.

## Introduction

The terms gender dysphoria and gender incongruence describe a heterogeneous group of individuals who express dissatisfaction with their anatomic gender and the wish to have the secondary sexual characteristics of the opposite sex.^1^ While not all transgender individuals suffer gender dysphoria, many do. In recent years, there have been significant advances in the understanding, management, and care of transgender persons. These developments encompass psychological, medical, and surgical approaches to therapy to help alleviate gender dysphoria. In addition, social and political changes over the past 35 years have brought more attention to this underserved and diverse population. In fact, in 2010, the World Professional Association for Transgender Health (WPATH) released a statement recommending the de-psycho-pathologization of gender nonconformity, stating that the expression of gender characteristics, including identities, which are not stereotypically associated with one's assigned sex at birth, is a common and culturally diverse human phenomenon that should not be judged as inherently pathological or negative.^2^

The WPATH developed the *Standards of Care* (SOC) to help provide the highest standards of care for transgender individuals. Since WPATH published the first version of SOC in 1979, the guidelines have been updated seven times, illustrating the complex and developing needs in caring for the transgender population. SOC state that the overarching treatment goal is “lasting personal comfort with the gendered self to maximize overall health, psychological well-being, and self-fulfillment.”^2^ Toward this end, gender confirmation surgery provides the appropriate physical morphology. Congruent genitalia allow an individual to experience harmony between one's body and self-identity, appear nude in social situations without violating taboos (i.e., health clubs, physician offices), and, in some states, have legal identification concordant with one's physical appearance.^3^

For many transfemales, a successful surgical result involves the creation of a natural-appearing vagina and mons pubis, which are sensate and functional. This includes removal of the stigmatizing scrotum, creation of feminine-appearing labia majora and minora, construction of a sensate neoclitoris, and development of adequate vaginal depth and introital width for intercourse. Additional desirable qualities include a moist appearance to the labia minora, clitoral hooding, and lubrication for intercourse.

Aside from genital reconstruction, breast augmentation, thyroid chondroplasty (tracheal shave), and facial feminization offer additional procedures designed to feminize one's appearance.

For transmen, as outlined by Professor Stan Monstrey and his team at the University of Ghent, (Belgium), phallic reconstruction should result in an aesthetic phallus with both tactile and erogenous sensations, the ability to void while standing, minimal morbidity of the surgical intervention and donor site, an aesthetic scrotum, and the ability to experience sexual satisfaction postoperatively.^4^

While phalloplasty represents the most complete genitoperineal transformation, it requires complex staged procedures, the use of tissue from remote sites resulting in scarring at the donor site, and increased risk of complications associated with urethral reconstruction and implantable prostheses. For these reasons, some individuals undergo metoidioplasty. This entails lengthening of the virilized clitoris and may be performed with, or without, urethral lengthening to allow for urination while standing.

In addition to genital surgery, chest surgery, involving bilateral subcutaneous mastectomies and contouring of the chest, is commonly performed in transmen. Chest surgery also includes repositioning and resizing of the nipple–areola complex when necessary.

## Hormone Therapy

Many, but not all, individuals with gender dysphoria desire hormone therapy to transition. Endocrinologists or primary care providers typically guide therapy. As with surgery, hormone therapy requires a tailored approach. Not all gender surgery (i.e., chest surgery) requires preoperative hormone therapy. However, some surgeries, such as metoidioplasty or phalloplasty, require adequate hormonal therapy to allow for clitoral virilization. Often times, third-party payers require documentation of 12 months of hormone therapy, or an explanation as to why a patient does not take hormones, before authorizing surgery.

## Preoperative Evaluation

The diagnosis of gender dysphoria is generally made by mental health providers who then refer individuals for surgical evaluation. Before performing gender confirmation surgery, the surgeon must verify that the diagnosis or gender dysphoria or gender incongruence is accurate. As noted by Dr. J.J. Hage, the surgeon remains responsible for any diagnosis on the basis of which he or she performs surgical interventions.^5^ Communication between the surgeon and mental health professional(s) is recommended. This serves to educate the surgeon and to aid with his or her understanding of each patient's unique needs. It also helps to prevent possible falsification of letters of recommendation. Communication should not be limited to the evaluation phase. It is the responsibility of the surgeon to communicate pertinent operative findings as well as postoperative instructions with the relevant members of the healthcare team.^6^ This includes mental health professionals, primary care providers, endocrinologists, mid-level practitioners, and other surgeons involved in an individual's care.

Several models for preoperative evaluation exist. For example, some centers follow an informed consent, or shared decision-making model, in which the healthcare provider and the patient make healthcare decisions together, taking into account best clinical practices and the preferences of the patient. However, most surgeons and third-party payers follow guidelines as provided in the WPATH SOC.

As per the WPATH guidelines, evaluation by behavior health professionals is central in preoperative assessment. These professionals can come from a variety of backgrounds, including psychology, psychiatry, social work, mental health counseling, nursing, or medicine, and should have specific and ongoing training in evaluating and caring for transgender individuals.^3^ The number of referral letters needed from mental health providers depends on the procedure requested. In general, nongenital surgery requires one referral letter, whereas genital surgery requires two referral letters.

Although multiple studies confirm the efficacy of surgery and low complication rates,^7,8,9–13^ the surgeon must actively investigate potential risk factors that may increase the risk of postoperative complications before proceeding with surgery. Based upon questionnaire data from 19 gender clinics in Europe and North America, conditions that could result in delay or denial of surgery included psychosocial instability, married status, substance abuse, chronic or psychotic illness, and antisocial behavior.^14^ In addition, in a retrospective review of 136 patients who underwent sex reassignment in Sweden, several preoperative factors were identified and reported to be associated with higher rates of unsatisfactory surgical outcomes. These included personal and social instability, unsuitable body build, and age over 30 years at operation. Additionally, in this study, adequate family and social support were noted to be important for postoperative functioning.^15^

Although understanding potential preoperative risk factors is important, their presence is not necessarily a contraindication to surgery. It must be emphasized that the SOC are not intended as barriers to surgery, but rather as a means of identifying patients who would benefit from surgical therapy.

In addition to mental health evaluation, additional input from a patient's primary care doctor or endocrinologist is useful. Documentation of hormone therapy, if applicable, and confirmation that a patient is medically fit for surgery are important before planning surgery.

Once the surgeon is satisfied that the diagnosis has been established, surgical therapy is considered. A preoperative surgical consultation is obtained, during which the procedure and postoperative course are described, the potential risks and benefits of surgery are reviewed, and the patient's questions are answered. Equally important is a discussion of the patient's expectations as well as an understanding of the limitations of surgery. In a study of 55 transgender patients treated in Belgium, De Cuypere et al.^9^ noted that transgender persons' expectations were met at an emotional and social level, but less so at the physical and sexual level. This occurred despite an indicated improvement in sex life and sexual excitement after reassignment surgery.^9^ Based upon these findings, it was recommended that discussion regarding sexual expectations be entertained before surgery.

If an individual decides to proceed with surgery, written documentation of informed consent should be included in the patient's chart.

## Gender Surgeries

### Transfemale genitalia surgery

Surgical conversion of the genitalia of transwomen has evolved since the use of skin grafts for creation of a neovagina in cases of vaginal agenesis.^16^ The use of pedicled penile and scrotal flaps was described over 40 years ago and remains the foundation for neovaginal construction.^17–19^ The vascular basis of these flaps is derived from one of two sources: (1) the femoral artery (deep and superficial external pudendal arteries) and (2) the internal pudendal artery (perineal branches).

Although a functional vaginoplasty is performed in a single stage, labiaplasty can be performed at a second surgical stage. Labiaplasty, which can be performed under local anesthesia as an outpatient procedure 3 months after vaginoplasty, creates a convergent anterior commissure and provides additional clitoral hooding. However, with recent trends toward hair removal of the mons, fewer individuals opt to proceed with the labiaplasty.

The surgical options for vaginoplasty consist of one of three options: penile inversion vaginoplasty, intestinal transplantation, or nongenital flaps. Most centers perform primary vaginoplasty with the penile inversion vaginoplasty using an anteriorly pedicled penile skin flap combined with a posteriorly based scrotal-perineal flap and/or skin graft. However, intestinal transposition, typically reserved for revision cases, is a first-line surgical therapy at some centers. The advantage of intestinal transposition is the creation of a vascularized 12–15 cm vagina with a moist lining. This may lessen the requirements for postoperative vaginal dilation as well as the need for lubrication during intercourse. However, the drawbacks of intestinal transposition include the need for an intra-abdominal operation with a bowel anastomosis and the potential for neovaginal secretions. Nongenital flaps are typically considered for reconstruction following oncologic resections, traumatic repair, or reconstruction following infection.

Regardless of the technique utilized, the author recommends that hormones are discontinued for ∼2 weeks out of concern for risk of venous thromboembolism (VTE). Some centers opt to continue hormones or change to the transdermal route before surgery; however, there are no studies evaluating clinical outcomes with these protocols. In addition, a preoperative bowel prep is administered. Before surgery, sequential compression devices are placed and intravenous antibiotics are administered. Following induction of general anesthesia, chemoprophylaxis for VTE is administered subcutaneously (either fractionated or unfractionated heparin, depending upon institutional policies), the patient is positioned in lithotomy, and an indwelling urinary catheter is placed.

### Penile inversion vaginoplasty

**FIG. 1.**
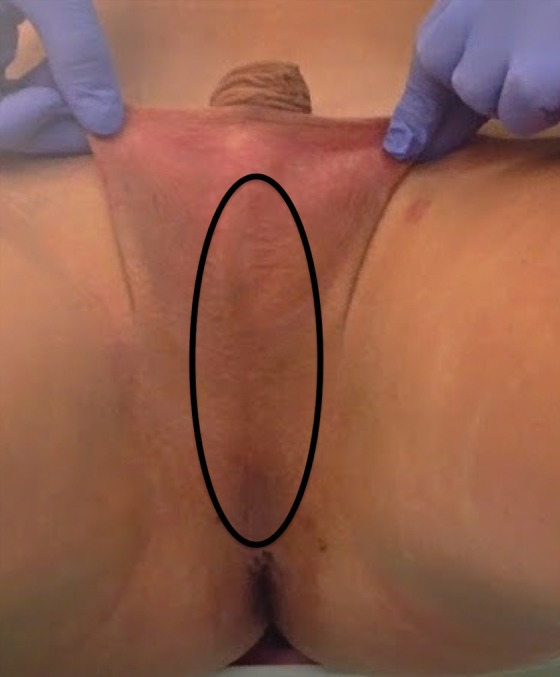
Approximate area of depilation for scrotoperineal flap.

Hair removal, whether by electrolysis or laser, is completed as thoroughly as possible from the penile shaft and central perineum and scrotum (Fig. 1) before penile inversion vaginoplasty. Preoperative depilation helps to prevent intravaginal hair growth. Adequate hair removal can take 3–6 months to complete and should not be performed within 2 weeks of surgery.

**FIG. 2.**
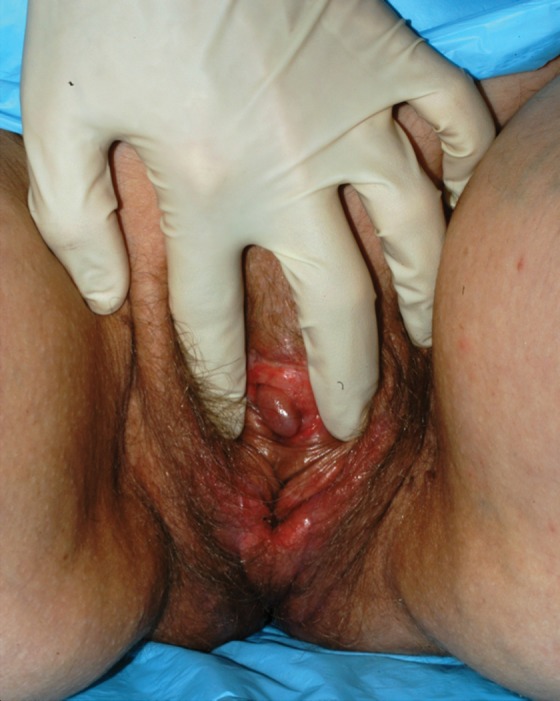
Postoperative penile inversion vaginoplasty.

Although a variety of technical modifications for penile inversion vaginoplasty are described in the literature, the penile disassembly and inversion technique utilizes the penile skin and a second, posteriorly based, scrotal-perineal flap to construct the vaginal cavity.^16^ The author's preferred technique is described herein (Fig. 2): the labia majora are formed from the lateral aspects of the scrotum, the neoclitoris is formed from the dorsal glans penis, and the labia minora are formed from the penile skin and urethral flap. The penile urethra is shortened, spatulated, and everted to create the neourethral meatus. Depending upon the length of the penis and previous surgical history (i.e., circumcision), skin grafts may be required for additional vaginal depth. Full-thickness skin grafts may be harvested from discarded portions of the scrotum. If this is insufficient, additional full-thickness skin grafts may be harvested with a Pfannenstiel incision.^20^ Alternatively, split-thickness skin grafts may also be harvested from the lower abdomen or mons region.

The postoperative care consists of a variable period of bedrest, during which a vaginal stent or packing is used to maintain the vaginal cavity. A urinary catheter remains in place until the vaginal packing is removed and ambulation is initiated, typically 5–6 days after surgery. Once the vaginal stent is removed, a regimen of vaginal dilation with a prosthesis is begun. Additionally, intermittent vaginal douching with a dilute povidone–iodine solution is performed to remove intravaginal debris. Vaginal intercourse may begin 6–8 weeks after surgery. An annual speculum and prostate examinations are also recommended.

Early postoperative complications include bleeding, infection, and delayed wound healing. Additional early or late complications include rectovaginal fistula, urinary stream abnormalities, inadequate vaginal depth or constricted introitus, partial flap loss, loss of neoclitoral sensation, and an unsatisfactory cosmetic appearance.

### Intestinal vaginoplasty

The advantage of intestinal transposition, especially in revision cases, is the provision of a reliable length of vascularized tissue with mucus secretion providing lubrication for vaginal intercourse. Intestinal transposition may utilize either the small or large intestine; however, the sigmoid colon is the most commonly used. The advantage of the sigmoid colon is the larger luminal diameter and less copious secretions compared with that of jejunum or ileum. Before performing the sigmoid vaginoplasty, a preoperative colonoscopy is performed so as to evaluate for pre-existing colorectal malignancies.

**FIG. 3.**
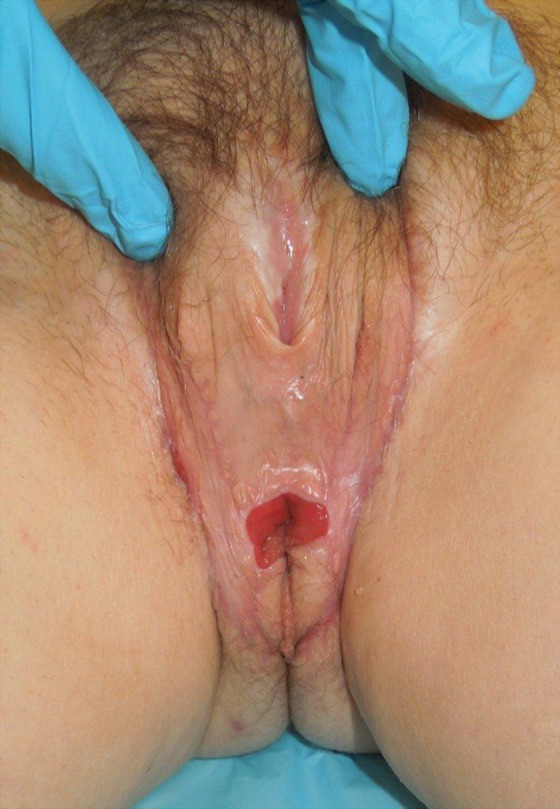
Postoperative intestinal vaginoplasty using sigmoid colon.

The sigmoid vaginoplasty (Fig. 3) is also performed in the lithotomy position in conjunction with general surgery. A combined abdominal and perineal approach is utilized and allows visualization and protection of the bladder and urethra anteriorly and the rectum posteriorly. Most recently, the harvest of the sigmoid colon was performed in a minimally invasive manner with the use of the operating robot and/or laparoscopic assistance. The sigmoid colon is harvested by the general surgery team, and the perineal dissection is performed concurrently by the plastic surgery team. A 12–15-cm segment of sigmoid colon is transferred in an isoperistaltic manner. The defunctionalized sigmoid colon is sutured to the introitus of the neovagina. Additionally, the mesentery of the defunctionalized sigmoid colon may be sewn to the pelvis to prevent torsion of the vascular pedicle. An end-to-end colonic anastomosis is performed, and the distal stump of the neovagina is separated from the colorectal anastomosis so as to reduce the risk of fistulization.

The neovagina is packed with nonadherent gauze, and the patient remains on bedrest with a urinary catheter for 1–2 days. Upon return of bowel function and oral intake, the patient is discharged from the hospital.

The potential drawbacks of intestinal-based flaps include secretions, most notably with the small intestine, and possible malodorous discharge with the large intestine. Additional concerns include the possibility of diversion colitis in the defunctionalized sigmoid colon as well as the risk of gastrointestinal malignancies. Finally, the colonic mucosa may be somewhat friable, and small amounts of postcoital bleeding may occur.

### Transfemale breast augmentation and facial feminization

Additional feminizing procedures include both breast augmentation and facial feminization. The goal of these procedures is to remove the secondary sexual characteristics and stigmata associated with the biological male appearance. The timing of these surgeries in relation to genital surgery may vary between centers as well as within individual centers. It is not uncommon for these feminizing procedures to be performed before genital surgery so as to improve an individual's sense of well-being.^7^

Following hormonal therapy, there is frequently some breast growth in the transwoman. However, the degree of breast growth is often inadequate, and individuals may continue to wear external prostheses or padded bras. As such, augmentation mammaplasty may be requested. Anatomic differences between the male and female chests are relevant with regard to implant selection, incision choice, and pocket location.^21^

**FIG. 4.**
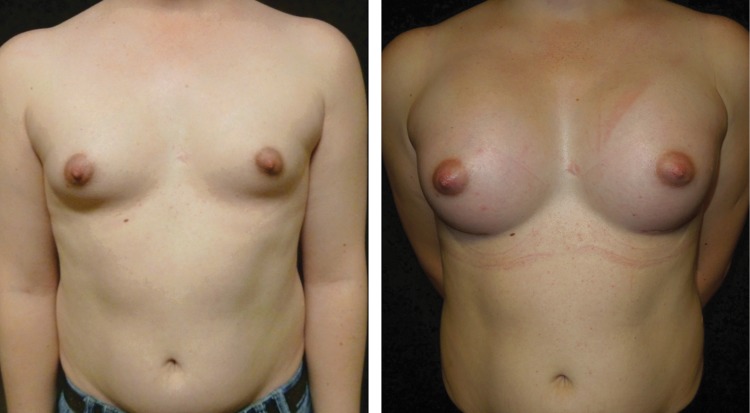
Breast augmentation in a transwoman.

The male chest is not only wider than the female chest but the pectoral muscle is also usually more developed. Furthermore, the male areola is smaller than the female areola, the distance between the nipple and inframammary crease is less, and there is less ptosis in the natal male breast, even after hormonal therapy.^22^ Based upon these characteristics, a larger implant is commonly chosen (Fig. 4). Pocket location and incision choice depend upon the individual and the degree of breast growth in response to hormonal therapy. A subglandular, subfascial, or subpectoral pocket may be used. Most often, silicone implants and, more recently, form-stable silicone implants are utilized.

In addition to breast augmentation, surgery to feminize the face of transwomen may be requested. A variety of characteristics have been identified as male and are often associated with the forehead, nose, malar region, mandible, and thyroid cartilage.

**FIG. 5.**
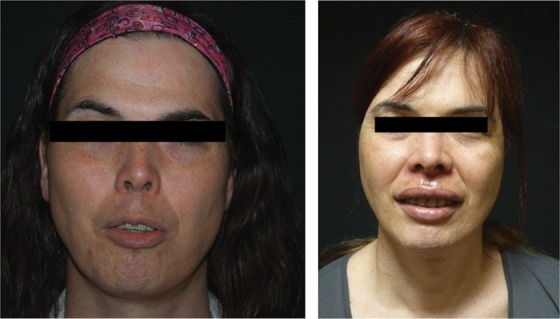
Frontal bone reduction with brow lift, upper lip shortening, and lip augmentation in a transwoman.

Because the female eyebrow is located above the supraorbital rim and has a more arched appearance than the male, typical procedures for facial feminization include a brow lift with advancement of the frontal hairline and frontal bone reduction (Fig. 5). Although the brow lift may be performed with an endoscope, reduction of the frontal bone and lateral brow, as well as the advancement of the frontal hairline, is facilitated with the open approach. Depending upon the thickness of the anterior table in relation to the degree of frontal bossing, craniofacial techniques may be employed for the desired correction.

**FIG. 6.**
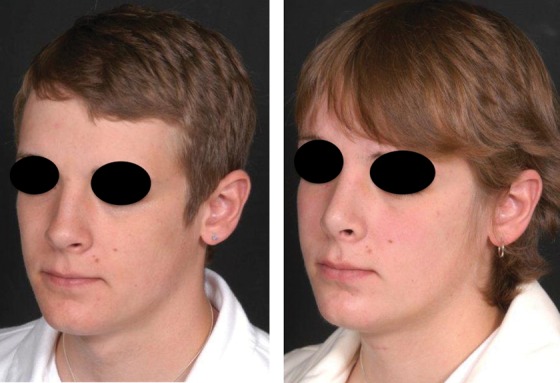
Rhinoplasty and thyroid chondroplasty in a transfemale.

A feminizing rhinoplasty typically involves dorsal hump reduction, cephalic trim, elevation of the nasal tip, and osteotomies to narrow the nasal pyramid (Fig. 6). Individuals may also request feminization of the chin and mandible. Based upon an individual's anatomy, either chin implants or osteoplastic genioplasty may be required. In addition, reduction of the masseter muscle or contouring of the mandibular angle may be performed through intraoral incisions. Other procedures, such as upper lip shortening, facelift, blepharoplasty, malar implants, hair transplantation, injectable fillers, and skin resurfacing, may also be performed.

Reduction thyroid chondroplasty may be requested to reduce the appearance of the Adam's apple or prominent thyroid cartilage (pomus Adamus) (Fig. 6). The procedure is typically performed as an outpatient under general or local anesthesia with sedation. The procedure is performed through a transverse incision in a naturally occurring skin crease. Following vertical division of the middle cervical fascia, the sternothyroid and thyrohyoid muscles are retracted laterally. On the posterior surface of the cartilage, subperichondrial dissection is performed inferiorly to the thyroepiglottic ligament. Identification of the insertion of the vocal cords may be facilitated with fiberoptic laryngoscopy by the anesthesthiologist.^23^ Resection of the thyroid cartilage is performed between the superior thyroid notch in the midline and the superior thyroid tubercle superolaterally.^24^

Voice surgery, designed to raise vocal pitch, may be requested by individuals following voice therapy, although its efficacy is debated.^25,26^ Hormonal intervention does not commonly affect vocal pitch and this may represent a residual stigma of masculinity. As such, various techniques to shorten the vocal cords, increase vocal cord tension, or reduce vibrating vocal cord mass may be performed.^27^

### Transmale chest surgery

Chest wall contouring is an important early surgical step in the process of gender confirmation for transmen and may help to facilitate transition. The goals of chest surgery include the esthetic contouring of the chest by removal of breast tissue and skin excess, reduction and repositioning of the nipple–areola complex, release of the inframammary crease, and minimization of chest scars.^28^

**FIG. 7.**
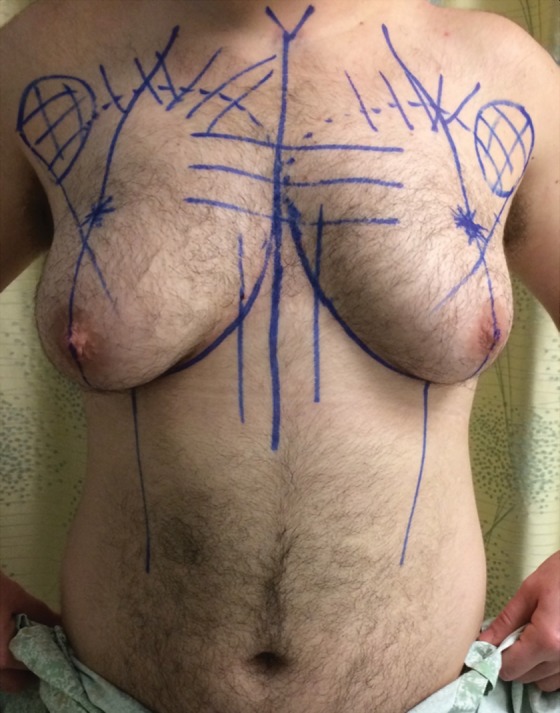
Preoperative transmale before chest surgery.

Chest surgery in transmen presents an aesthetic challenge due to breast volume, breast ptosis, nipple–areola size and position, degree of skin excess, and potential loss of skin elasticity (Fig. 7). Choice of incision is largely determined by degree of breast ptosis and skin quality/elasticity, as well as position of the nipple–areola complex.^29^ Incisions may range from a periareolar incision in small breasts with a small areola and good skin elasticity, to circumareolar incisions, to transverse inframammary crease incisions with free nipple grafts. Liposuction may be used as an adjunct to excisional techniques. In general, the nipple areola is positioned just medial to the lateral border of the pectoralis major muscle, ∼2–3 cm above the inferior insertion of the pectoralis major muscle. Postoperative management includes drains and elastic compression. Secondary revisions related to the scar and or nipple–areolar complex are not uncommon.

### Transmale genitalia surgery

The goal of genital surgery in transmen requires individualization. Surgery may range from clitoral release (metoidioplasty) with or without urethral lengthening (to allow for voiding while standing) to a phalloplasty, capable of sexual penetration.^29^

**FIG. 8.**
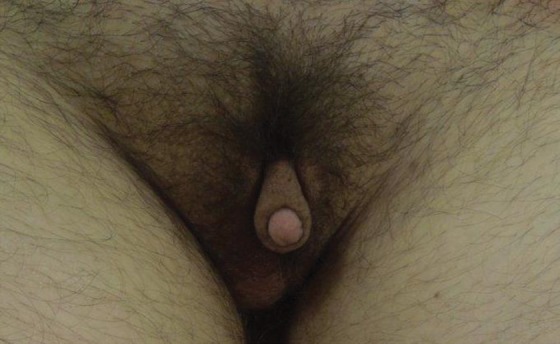
Postoperative metoidioplasty in a transman.

Metoidioplasty, described in 1996 by Hage, has been offered as an alternative to microsurgical or pedicled flap phalloplasty in transmen (Fig. 8). The procedure entails lengthening the hormonally hypertrophied clitoris by release of the suspensory ligament and resection of the ventral chordee and lengthening of the female urethra with the aid of labia minora and/or vaginal musculomucosal flaps.^29^ Additionally, buccal mucosa grafts have been utilized to aid with urethral extension. Urethral reconstruction is the major challenge associated with metoidioplasty, and most complications involve either urethral fistulae or strictures.

The operative technique may involve concomitant removal of the female genitalia (vaginectomy) in addition to metoidioplasty.^30^ Most often, a hysterectomy and oophorectomy are performed before the metoidioplasty. A caudally based anterior vaginal wall flap incorporating the muscularis of the ventral vaginal wall may be used. This flap can be used to reconstruct the fixed portion of the neourethra.^31^ The clitoral shaft is degloved and released by detaching the suspensory ligament from the pubic bone. On the ventral aspect of the clitoris, the urethral plate is dissected from the clitoral bodies. The urethral plate is divided so as to release the ventral clitoral curvature, allowing straightening and lengthening of the clitoris.^30^ Additional lengthening of the urethra is performed with flaps developed from the labia minora.

Scrotoplasty, constructed with bilateral labia majora flaps, may be performed at the time of metoidioplasty. Testicular implants are placed at a secondary surgical procedure so as to reduce the risk of infection and urethral complications.

**FIG. 9.**
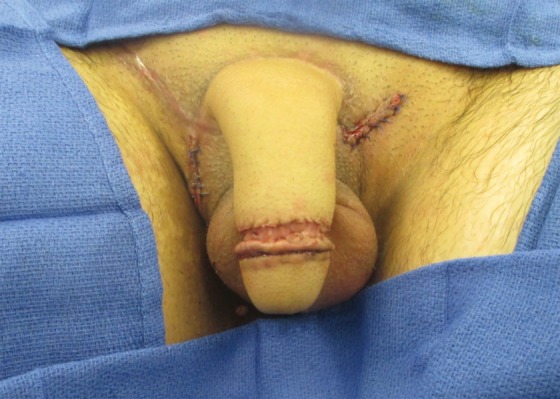
Postoperative phalloplasty in a transman.

Phalloplasty represents the most complete genitoperineal transformation for transmen (Fig. 9). Phalloplasty techniques may be divided into pedicled flaps and free flaps. Pedicle flaps transfer tissue, typically of the thigh, groin, or lower abdomen, to reconstruct the penis, while free flaps involve the microsurgical transfer of tissue from a remote location.

The most common technique for phallic reconstruction is the radial forearm-free flap. This procedure transfers tissue, including blood vessels and nerves, from the forearm to reconstruct the penis and urethra. This flap allows single-stage reconstruction of a sensate phallus and glans penis. Potential drawbacks of this technique include the visibility of the donor site on the forearm and the need for microsurgical skills.

Perhaps the next most common technique for phalloplasty is the use of tissue from the thigh, known as the anterolateral thigh flap. Similar to the forearm technique, tissue, including nerves, may be transferred from the thigh to construct the penis. Depending upon the individual's distribution of subcutaneous fat, a second flap may be required for urethral reconstruction.

For both the forearm and thigh techniques, the urethra is formed by a skin-lined tube. As such, preoperative electrolysis may be required for depilation of the urethra.

Additional phalloplasty techniques include the use of tissue from the back, known as the musculocutaneous latissimus dorsi flap. One notable downside of this flap is the lack of a sensory nerve to the tissue that will be used to construct the phallus.

### Surgery for gender nonconforming/expansive individuals

It has been increasingly recognized that many individuals with gender dysphoria do not see themselves in traditional male or female categories. Gender nonconformity or gender expansive describes a difference between an individual's gender identity, role, or expression and that of cultural norms. For these individuals, varying degrees of hormone therapy and surgical intervention may be helpful for allowing them to become their true selves. Similar to other transgender persons, a customized multidisciplinary approach is necessary to provide appropriate medical and surgical care to gender nonconforming individuals.

## Measuring Outcomes

The field of gender confirming surgery has grown with increased awareness, need, and third-payer coverage. However, there are still no formal training programs or specific board certifications for surgeons performing gender confirming surgery. This will likely change in the coming years. Currently, surgery is performed by reconstructive plastic surgeons, urologists, and gynecologists, often in a collaborative approach. In addition, methods to accurately measure patient outcomes are still under development. Working groups are currently participating with the National Institutes of Health to develop systems to capture and report these important data.

## Conclusion

Significant advances in social, medical, and surgical care have led to improved access to care for transgender individuals. Gender surgery presents one facet of a complex multidisciplinary approach to allow transgender individuals to become who they know themselves to be. Continued collaboration between the surgeon, mental health professional, and medical physician in the framework provided by the WPATH SOC guidelines is important in providing quality care to transgender individuals. In addition, continued research focused on objective parameters and reporting of outcome data will foster innovation and continued improvements in surgical techniques.

## Abbreviations Used

SOC: *Standards of Care*
VTE: venous thromboembolism
WPATH: World Professional Association for Transgender Health
